# Summative assessment of 5^th^ year medical students’ clinical reasoning by script concordance test: requirements and challenges

**DOI:** 10.1186/1472-6920-12-29

**Published:** 2012-05-09

**Authors:** Paul Duggan, Bernard Charlin

**Affiliations:** 1 Discipline of Obstetrics and Gynaecology, The University of Adelaide, Frome Rd, Adelaide, South Australia, 5000, Australia; 2 CPASS, direction de la recherche, Faculté de Médecine, Université de Montréal, CP 6128, Succursale centre- ville, Montréal, Québec, H3C 3 J7, Canada

## Abstract

**Background:**

The Script Concordance Test (SCT) has not been reported in summative assessment of students across the multiple domains of a medical curriculum. We report the steps used to build a test for summative assessment in a medical curriculum.

**Methods:**

A 51 case, 158-question, multidisciplinary paper was constructed to assess clinical reasoning in 5^th^-year. 10–16 experts in each of 7 discipline-based reference panels answered questions on-line. A multidisciplinary group considered reference panel data and data from a volunteer group of 6^th^ Years, who sat the same test, to determine the passing score for the 5^th^ Years.

**Results:**

The mean (SD) scores were 63.6 (7.6) and 68.6 (4.8) for the 6^th^ Year (n = 23, alpha = 0.78) and and 5^th^ Year (n = 132, alpha =0.62) groups (p < 0.05), respectively. The passing score was set at 4 SD from the expert mean. Four students failed.

**Conclusions:**

The SCT may be a useful method to assess clinical reasoning in medical students in multidisciplinary summative assessments. Substantial investment in training of faculty and students and in the development of questions is required.

## Background

Script theory explains how physicians progressively acquire knowledge adapted to their clinical tasks [1,2]. The Script Concordance Test (SCT) is a relatively new tool [3] designed to measure a specific but crucial element of clinical reasoning: clinical data interpretation [4]. One significant characteristic of the SCT format is that it allows testing in ill-defined contexts that are often typical of clinical practice. Publications reporting its use in assessment in domains such as radiology, neurology, radio-oncology, surgery or emergency pediatric medicine have indicated noticeable psychometric qualities [5-9]. Tests were reliable, with Cronbach’ s alpha coefficient of internal coherence reaching values of 0.75 or more in one hour of testing time [6-9], and showed construct validity, with consistent findings of statistically linear progression of scores with clinical experience [10,11].

These studies have been realized with volunteer participants of differing level of clinical expertise. While a few studies, such as the one of Collard et al., [12] have assessed reasoning among same level medical students in specific domains, there are not yet reports of SCT used as summative exam of clinical reasoning across the multiple domains of a medical curriculum. Undertaking such an enterprise raises many issues such as item production, scoring with panels from different disciplines, test administration, setting of passing scores.

## Context

At the University of Adelaide, assessment of clinical competence in the 5^th^ year of the six-year medical program comprises a three-hour multiple choice question (MCQ) paper, an eighteen-station OSCE and a tool specifically aimed at measurement of clinical reasoning. A three-hour modified essay question paper (MEQ) was used for that purpose until 2008 when an assessment review [13] established that this exam was predominantly testing factual recall and that the faculty was having difficulty with question writing and marking. Beyond that, when a modified Angoff standard setting was undertaken to move from norm-referencing to criterion-referencing, it was found that some disciplines had unrealistically high expectations and therefore an unacceptable rate of failure.

Since then, while other dimensions of clinical competence are still assessed with the MCQ paper and the OSCE, the MEQ has been replaced by a three-hour SCT to assess clinical reasoning. This choice was based on a series of arguments: documented reliability [6-9], capacity to test reasoning in situations in which there is a degree of uncertainty and in which no single response applies [4], computerized marking and rapid turn around of results.

## Purpose

To report the steps used to build a test for summative assessment in a medical curriculum including the steps taken to construct and score on line a test covering a wide range of domains; to report the qualities and drawbacks of the SCT.

## Methods

### Structure, production and scoring of the SCT cases and questions

The SCT format is shown in Table 1. This provides a clinical scenario (case), a hypothesis or plan of action based on the scenario, and some additional information that may or may not have an effect on the hypothesis or plan. Each scenario is followed by up to 5 questions. For each of them, the participant selects the single best Likert response that describes the effect of the additional information that has been given. In contrast to many conventional forms of testing, there are no single correct answers in SCT questions; several responses to each question may be considered acceptable. The examinee’s response to each question is compared with those of a panel. Credit is assigned to each response based on how many of the experts on the panel choose that response. A maximum score of 1 is given for the response chosen by most of the experts (i.e., the modal response). Other responses are given partial credit, depending on the fraction of experts choosing them. Responses not selected by experts receive zero [4].

**Table 1:** An example of a SCT case vignette with two questions

	If you are considering the following investigation …	and then you find …	you would then consider the proposed investigation to be …
Q1	A ventilation-perfusion scan to rule out pulmonary embolism	Her chest X-ray demonstrates areas of collapse	∘ much less useful
			∘ slightly less useful
			∘ neither less nor more
			∘ useful
			∘ slightly more useful
			∘ much more useful
Q2	An arterial blood gas	Her oxygen saturation whilst breathing room air is 96%	∘ much less useful
			∘ slightly less useful
			∘ neither less nor more useful
			∘ slightly more useful
			∘ much more useful

Questions were written by discipline-based experts and reviewed independently by at least one other person before being placed on a web site for administering the SCT. Question writers were also members of the panel in their discipline. The questions were written over a period of approximately 9 months.

### Web-based and computer-assisted activities

To facilitate item management (use of multimedia, item banking, item verification, and item selection) and communication to faculty and students, development of the test was web-based. This was achieved by a partnership with the University of Montreal, which has a web site dedicated to SCTs http://www.cpass.umontreal.ca/sct.html.

### Expert reference panels (ERPs)

In contrast with published SCT studies, where all test items belonged to single domains, in our context items belonged to several domains. We therefore had to create distinct panels for each domain. An expert reference panel (ERP) is a discipline-based group of experts who independently sit the same questions under similar conditions to candidates (limited time and no access to reference material or other people). We established ERP’s in the disciplines of General Practice, Medicine, Obstetrics and Gynaecology, Paediatrics, Pathology, Psychiatry and Surgery with between 10–16 members in each of the 7 panels. It has been recommended that panels have at least 10 members to get reliable scores [11]. The number of members in each panel reflected both the size of each discipline and the level of interest in participation in the SCT by the various disciplines. The fist author, who had previous experience in this, trained panel members in script theory and how to write and answer SCT questions [14]. Training was undertaken in workshops and was also available on-line in a public web page http://www.fpcmed3.umontreal.ca/www.health.adelaide.edu.au/ with additional material in a secure web location only accessible to ERP members.

Clinical cases and their questions were all placed on the secure web site for experts to answer. Several cases were multidisciplinary (e.g. Medicine and Surgery) and in those instances answer keys were produced using the answers from all of the participating panels.

### Selection of questions

The SCT paper was structured to ensure a spread of modal answers of the ERP across the 5-point range. An assessment panel analyzed the potentially suitable questions for relevance to the curriculum, fairness for the level of candidate, appropriateness of the modal response in relation to current best evidence, and appropriateness and clarity of language. We excluded questions that appeared only to be testing factual recall or that were otherwise inappropriate. The final selection was then determined in relation to the needs of the summative assessment as a whole and following our assessment blueprint, taking in to account the topics covered in the MCQ and OSCE.

### Analysis of ERP responses

When there was a single outlier in the ERP responses, that person’s response for the question was deleted. This was done as we had received feedback from experts that in some cases they had accidentally selected the opposite Likert response to what they intended, but had realized this too late to undo the selection (a problem with our “unforgiving” on-line technology). This problem was reported by experts only when the most extreme Likert responses were selected - for example, accidentally selecting a phrase like “extremely inappropriate” when meaning to select “extremely appropriate”. We felt this was a reasonable explanation for single outliers, and that deleting a single extreme outlier was more appropriate than retaining it, noting that the effect on the scoring key would be negligible either way.

The few questions with a single Likert response selected by all panel members, were excluded as we considered these did not reflect uncertainty. Questions in which there was reasonable expert concordance (a distribution of one Likert scale point around the modal response after deletion of obvious outliers) were considered for inclusion. Answers kept after these analyses were used to build the answer key.

Questions categorized as “discordant” and not suitable for inclusion followed two patterns: a bimodal-type response with disjointed modes or no clear mode with answers across the range. Performances of ERP members on the whole test were computed, kept anonymous and used to compute panel mean and standard deviation. These data served to determine students’ passing score.

### Training of medical students

Students were informed early in the year that the MEQ paper was being replaced with the SCT. The students were given examples of SCT questions and strategies on answering these questions in special lectures. The lectures were supplemented by on-line material (http://www.cpass4.umontreal.ca/www.health.adelaide.edu.au/). This on-line material included a discussion of why the modal response was the preferred response for the sample questions.

### Trial run with 6^th^ year volunteers

There is no formal examination in 6^th^ Year in our program. 23 volunteers from the 2008 6^th^ Year cohort sat the SCT in advance of its deployment in the 5^th^ Year assessment in November. Goals of this trial run were to identify unforeseen problems in advance of the deployment of the 5^th^ Year paper and to use results as a reference to establish the passing score. Ethics approval was obtained from the University of Adelaide Ethics Committee for the 6^th^ Year trial run.

### Determining grading and pass/fail criteria

A multidisciplinary group of senior academics met before deployment of the 5^th^ Year paper to determine pass/fail criteria. The Adelaide medical program has competency-based assessments and issues a non-graded pass in its official results. However, informal grades that do not form part of the academic record are also provided as a guide to candidates (Table 2). A pass is awarded for A-C grades. ERP members score on average about 80% in SCT questions with a relatively small SD of about 5% (Table 3). Because experts are discipline-based and candidates are not, we calculated the weighted average mean and weighted average standard deviation of the expert’s scores. For example, if Discipline A contributed 70 of the questions and Discipline B 30 then the formula for the weighted average mean is (mean A x 0.7 + mean B x 0.3).

**Table 2:** **Grade descriptors, calculations and allocations for the 158 question 5**^**th**^**Year SCT**

**Grade and description**	**Relationship to expert score**	**Range for each grade**	**n students**
A Above expected competency for the year level	Within −2 SD	68+	44
B Clearly at expected competency for the year level	Within −3 SD	62-67	61
C Just reaches expected competency for the year level	Within −4 SD	56-61	23
D Below expected competency for the year level	Within -5SD	51-55	4
E Far below expected competency for the year level.	Anything less	<51	0

**Table 3:** Mean (SD) for expert reference panels by discipline for the 158 question SCT

**Experts' data**	**Medicine**	**O&G**	**Paediatrics**	**Pathology**	**Psychiatry**	**Surgery**	**Weighted average/total**
N questions	38	37	14	3	25	41	158
Mean	82	71	84	81	77	84	79.3
SD	5.6	5.8	6.3	18.1	6.3	4.4	5.7

We reasoned that a 5^th^ Year student scoring within 2SD of the weighted expert mean was performing to the level of an expert and hence above expected competency (“A” grade). The determination of the critical pass/fail cut point was more problematic. We made the assumption that our 5^th^ Years would perform in a similar way to our 6^th^ Year volunteers. We considered models that related the cut point for the 6^th^ year data to standard deviations (part or whole) from the expert mean and chose the model shown in Table 2, with the result that 4 SD below panel mean was set as the pass mark for the 5^th^ Year paper.

### Test administration

For students the test was administered as a paper-based test as the University does not yet have the facilities to administer in large numbers on line testing under examination conditions. Answer sheets were scanned and optical character recognition software used to produce an Excel spreadsheet of raw data. Student scores were computed from the raw data for the 51 cases/158 SCT questions with reference to the answer key derived from the ERPs using an array “look up” function in Excel. All students completed 6 additional questions at the end of the SCT to evaluate their experience of the test and these additional data were expressed as a percentage of respondents in each category for each variable. The entire assessment was run over three hours.

### Statistical analysis and estimating reliability of the SCT

Results of the SCT were correlated the MCQ and OSCE using the Pearson correlation function. Cronbach alpha statistics were computed with SPSS (version 16.0 for Mac, SPSS Inc Chicago). Reliability was estimated with the Cronbach alpha coefficient and by Rasch modeling (Winsteps® Rasch measurement version 3.69.1.16, copyright© 2009 John M Linacre, http://www.winsteps.com).

## Results

A total of 198 clinical cases containing 832 questions were initially produced. After review, 436 questions (52%) from the 198 cases were considered suitable for undergraduate assessment by our criteria. After the selection phase, the 5^th^ Year SCT paper comprised 51 cases and 158 questions (from 2–5 questions per case) from all contributing clinical disciplines.

23 6^th^ Year students volunteered to sit the SCT, to assist with standard-setting, and scored a mean (SD) of 63.6 (7.6). 132 5^th^ Year students sat the identical test as a summative test about 1 month later. The mean, standard deviation, minimum and maximum scores for the 5^th^ Year examination were 68.6, 4.8, 54.5 and 75.5, respectively. Four of the 132 5^th^ Year students had scores below 4 standard deviations from the panel mean and so failed the SCT (failure rate: 3%). The results for the 5^th^ Year and 6^th^ Year cohorts were statistically significantly different (p < 0.05).

**Figure 1:**
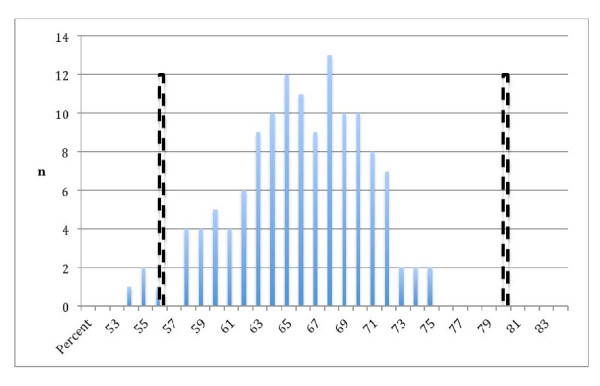
**Frequency distribution of scores of 5**^**th**^** Year medical students sitting a 158 questions Script Concordance Test.** Solid bars show students’ scores. Broken bars show the mean score of experts in the same test and the pass/fail cut point of >4 SD below the mean of the experts’ scores in the same test.

The spread of grades in the 5^th^ Year SCT is shown in Table 2 and the frequency distribution of the scores is shown in Figure 1.

The value for the Cronbach alpha coefficient was 0.78 for the 6^th^ Year volunteers and 0.62 for the same assessment in the 5^th^ Year cohort. Rerunning this calculation for the 5^th^ Year cohort with stepwise deletion of data from individual disciplines did not increase the alpha statistic. Cronbach alpha was 0.76 after removal of 40 items with a negative item-total correlation. Optimizing the Cronbach alpha in this fashion resulted in a small change in the mean and SD of the scores and an alteration in the spread of grades but no change to the failure rate. Four of the 132 5^th^ Year students had scores below 4 standard deviations from the panel mean and so failed the SCT (failure rate: 3%).

Data were analyzed using the information function in Rasch modeling. This analysis indicated that the critical pass/fail cut point was very close to the point of maximal reliability of the test but also reduced significantly as scores increased to the right of the cut point and was low at higher scores.

**Figure 2:**
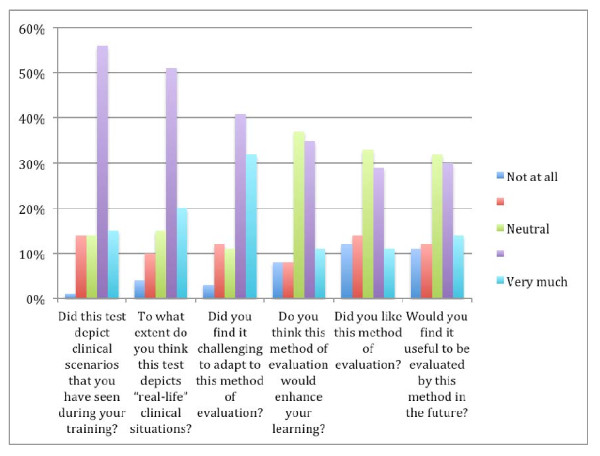
**Evaluation of the student experience of sitting the 5**^**th**^** Year SCT.**

The results of the evaluation of the student experience with the SCT are shown in Figure 2. The majority of students had positive views regarding the “real life” depiction of the test scenarios and the relevance of the test to student experience of clinical practice. The majority of students also found it challenging to adapt to this new method of assessment.

The results of the SCT were weakly-to-moderately correlated with the results of the MCQ (r = 0.5) and OSCE (r = 0.41).

## Discussion

This paper reports the implementation of the SCT format in our school as a mandatory test of clinical reasoning in medical students. The test format was well accepted. Conversely, student opinion of the enjoyment and educational value of the test was mixed. Students’ scores were widely spread and we found the distribution of scores to approximate what we expected of a 5th year cohort. It is reasonable to expect students in the 5^th^ year of a program to be performing well and high failure rates are hard to justify.

The failure rate of 3% was lower than the expected rate of 5% for the MEQ assessment, which was replaced by the SCT. Setting a passing score must result from a transparent, reproducible, objective, and defensible process [15], but ultimately standard setting is arbitrary. Some methods of standard setting have delivered pass marks that vary substantially between different universities for the same questions [16]. Also, there may be greatly different results within the same group of examiners depending on the method used [17]. Hypothetical prediction by experts of borderline performance, as is commonly employed in standard setting, seems intrinsically less fair or robust than correlating student performance with the real performance of a reference group in the same test as done with SCT. Performance of candidates can be related to the performance of reference panel of experts, using panel standard deviation as a yardstick [18]. However, it is also possible that results would have been different with different members in the reference panels. The approprieteness of this method of standard setting needs to be further explored and further research in alternative methods of standard setting, for example, as described by Collard et al., [19] is required.

Our group of senior examiners determined that 5^th^ year medical students who scored within 2 SD of experts in the same test were performing above expectations. The setting of the crucial pass/fail cut point at 4SD below the expert mean score occurred after detailed consideration of the performance of a volunteer 6^th^ year cohort in the same assessment. We had expected that the 6^th^ Year and 5^th^ Year groups would perform to a similar level, whereas the 5^th^ Year group performed significantly better, by 5%. Purely by chance, based on academic rank in their 5^th^ year assessments, the 6^th^ Year sample appeared to be representative of their cohort (data not shown). The difference in results in the SCT is probably best explained by differences in preparation - there were no stakes for the 6^th^ Year volunteers contrasted with high stakes for the 5^th^ Years.

The use of a new test format implied having to develop a bank of new questions, requiring the input of more members of our faculty than had been previously required for our MEQ. In addition, training of faculty members and students was required in this new method of assessment. The partnership we established with the University of Montreal to share web facilities has considerably eased this workload, especially for undertaking ERP work and for training on line. These web facilities also allow collaboration with other Australian universities in item banking, test administration and research.

Writing good SCT questions has proved not to be easy. This is true of any other type of question. The reasons for SCT questions being unsuitable are often not obvious. Firstly, the questions should not only be testing factual recall. Secondly, the selection of the terms in the Likert responses needs careful consideration in order to achieve a spread of modal responses across the 5-point range for the assessment as whole. For example, many colleagues do not feel comfortable with selecting extreme descriptors such as “essential” or “absolutely contraindicated”. We recommend avoiding such extreme descriptors and to provide a scale on only one dimension – such as “more probable, much more probable”. Thirdly, a surprising number of questions that appeared to be good questions were rejected due to discordance in the expert reference panel. We are still evaluating this and are unsure if those questions are “good” or “bad” and whether they can be used in assessment. In subsequent multidisciplinary reviews we established, whilst in some cases experts have simply made a mistake in selection of a Likert response (data entry error), that is not the commonest reason for expert discordance. We have found in some cases the question is ambiguous or has some other fault that has not been detected by its author and our original review panel. However, it is also apparent that for some questions some “experts” are simply wrong. For example, question 1 of Table 1 was excluded from consideration because of a scoring distribution that equally rewarded responses that indicated that a ventilation-perfusion scan was both more and less useful in the investigation of suspected pulmonary embolism in the presence of an abnormal chext Xray. In a post hoc review of this question it became apparent that some experts were simply not aware that the diagnostic accuracy of a ventilation-perfusion scan is lower in the presence of the specified abnormality on chest Xray, and that the alternative investigation of a spiral contrast CT scan was then the local gold standard in that situation. Thus, we have what appears to be a usable question on investigation of suspected pulmonary embolism that has a bimodal distribution that, if used, would award significant partial credit (up to 0.87 of a full mark) to unacceptable responses. We think this apparent lack of expertise in “experts” is partly a reflection of subspecialisation within disciplines and partly a reflection of some experts being out of touch in areas peripheral to their specific interests. This is an important observation and not only for undergraduate assessment. For now we have dealt with this problem by not using those questions. More research is needed on questions in which there is expert discordance.

This, of course, raises the question of who should be in expert reference panels. It may be that generalists are more appropriate members of panels when it comes to assessing medical students. In the traditional specialties, generalists as opposed to subspecialists appear to be more able to answer a broader range of questions. We have preliminary data (not shown) to suggest that general practitioner panels will score around the same mean as a specialist panel but with a wider SD. In relation to our example of the optimal investigation for suspected pulmonary embolism, we speculate that recent medical graduates would have no problem in answering that question in a manner that would be acceptable for its inclusion in assessment. We are currently undertaking further research in this, including with a reference panel of recent medical graduates.

Perhaps the strongest evidence of the validity of our SCT relates to the extensive process involved in developing and selecting the questions. This started with question writing by an expert with the question vetted by a single colleague before submission for ERP work, the ERP work itself that would appear to “weed out” most unsuitable questions (because we excluded questions in which experts were discordant in their responses), and a subsequent review by an experienced committee of examiners before deployment of the questions.

Based on the literature, we were expecting a Cronbach alpha of around 0.8 for our SCT. An alpha of 0.78 was computed for the 158 questions sat by the 23 6^th^ Year students. We were surprised then to compute an alpha of only 0.62 on the same questions in our 5^th^ year cohort. We, therefore, recalculated alpha after eliminating items with a negative item:total correlation, which of course improved the statistic, but on these data had surprisingly little effect on the results. In retrospect, we believe that identifying items with a negative item:total correlation should encourage a closer look at the items, for item writing flaws, but is not an indication to remove them unless flaws are uncovered.

There was no formal collection of perceptions of faculty on the new test format. Test preparation took a lot of energy from faculty, but the general impression was that the format induced reasoning activities that are closer to the reality of practice than our MEQ, the format formerly used. As all students, ours generally don’t like changes in the assessment system and this was reflected in mixed student evaluations of the experience of the new SCT. Since then we have introduced the SCT in earlier years of the medical program and by the later years our students are now very familiar with the SCT. Reports to faculty on the SCT by student representatives are now favourable.

## Conclusions

### What this study brings

A description of steps demanded by an SCT when used for same level students and on different domains of medicine

A proposition to set grades and passing score at predetermined distances of the mean of experts having taken the same test as students.

The use of the SCT format for summative evaluation of clinical reasoning achievement raises a series of research issues. However, it appears as a promising method of testing that we have now adopted for high stakes assessment in our curriculum.

## Abbreviations

ERP: Expert reference panel; MCQ: Multi choice question; MEQ: Modified essay questions; OSCE: Objective structured clinical examination; SCT: Script concordance test; SD: Standard deviation.

## Misc

Paul Duggan and Bernard Charlin contributed equally to this work

## Competing interests

The author(s) declare that they have no competing interests.

## Authors' contributions

Both authors contributed to the conception and design of the study and the drafting of the manuscript. PD performed the data analysis. Both authors read and approved the final manuscript.

## Pre-publication history

The pre-publication history for this paper can be accessed here:

http://www.biomedcentral.com/1472-6920/12/29/prepub
